# Delaying ACL reconstruction and treating with exercise therapy alone may alter prognostic factors for 5-year outcome: an exploratory analysis of the KANON trial

**DOI:** 10.1136/bjsports-2016-097124

**Published:** 2017-05-17

**Authors:** Stephanie R Filbay, Ewa M Roos, Richard B Frobell, Frank Roemer, Jonas Ranstam, L Stefan Lohmander

**Affiliations:** 1 Arthritis Research UK Centre for Sport, Exercise and Osteoarthritis, Nuffield Department of Orthopaedics, Rheumatology and Musculoskeletal Sciences, University of Oxford, Oxford, UK; 2 Research Unit for Musculoskeletal Function and Physiotherapy, Institute of Sports Science and Clinical Biomechanics, University of Southern Denmark, Odense, Denmark; 3 Department of Clinical Sciences Lund, Orthopaedics, Lund University, Faculty of Medicine, Lund, Sweden; 4 Department of Radiology, University of Erlangen-Nuremberg, Erlangen, Germany; 5 Quantitative Imaging Center (QIC), Department of Radiology, Boston University School of Medicine, Boston, Massachusetts, USA; 6 Independent Statistician, Mdas AB, Ystad, Sweden

**Keywords:** exercise therapy or rehabilitation, arthroscopic surgery, meniscus, cartilage, pain, quality of life

## Abstract

**Aim:**

Identify injury-related, patient-reported and treatment-related prognostic factors for 5-year outcomes in acutely ACL-ruptured individuals managed with early reconstruction plus exercise therapy, exercise therapy plus delayed reconstruction or exercise therapy alone.

**Methods:**

Exploratory analysis of the Knee Anterior Cruciate Ligament, Nonsurgical versus Surgical Treatment (KANON) trial (ISRCTN84752559). Relationships between prognostic factors (baseline cartilage, meniscus and osteochondral damage, baseline extension deficit, baseline patient-reported outcomes, number of rehabilitation visits, graft/contralateral ACL rupture, non-ACL surgery and ACL treatment strategy) and 5-year Knee Injury and Osteoarthritis Outcome Score (KOOS) pain, symptoms, sport/recreation and quality of life (QOL) scores were explored using multivariable linear regression. Estimates were adjusted for sex, age, body mass index, preinjury activity level, education and smoking.

**Results:**

For all participants (n=118), graft/contralateral ACL rupture, non-ACL surgery and worse baseline 36-item Short-Form Mental Component Scores were associated with worse outcomes. Treatment with exercise therapy alone was a prognostic factor for *less* knee symptoms compared with early reconstruction plus exercise therapy (regression coefficient 10.1, 95% CI 2.3 to 17.9). Baseline meniscus lesion was associated with worse sport/recreation function (−14.4, 95% CI −27.6 to –1.3) and osteochondral lesions were associated with worse QOL (−12.3, 95% CI −24.3 to –0.4) following early reconstruction plus exercise therapy. In the same group, undergoing additional non-ACL surgery and worse baseline KOOS scores were prognostic for worse outcome on all KOOS subscales. Following delayed reconstruction, baseline meniscus damage was a prognostic factor for *less* pain (14.3, 95% CI 0.7 to 27.9). Following exercise therapy alone, undergoing non-ACL surgery was prognostic for worse pain.

**Conclusions:**

Treatment-dependent differences in prognostic factors for 5-year outcomes may support individualised treatment after acute ACL rupture in young active individuals.

**Trial registration number:**

Current Controlled Trials ISRCTN84752559.

## Introduction

In 1983, one of the first literature reviews discussing surgical and non-operative management for ACL rupture concluded that ‘in the future, we hope to be able to discern more accurately which acute ACL ruptures need surgical treatment’.^1^ Thirty-three years later, evidence-based recommendations to guide optimal selection of non-operative or surgical management strategies for the acutely ACL-injured patient have not been established. Systematic reviews have found similar long-term outcomes (physical activity levels, pain, symptoms, knee osteoarthritis and quality of life (QOL)) following ACL reconstruction and non-operative management of ACL rupture, although most studies have been of poor methodological quality and very few randomised controlled trials (RCTs) exist.^2–5^ The only high-quality treatment RCT of the ruptured ACL, the KANON trial, found no difference in patient-reported, structural or functional outcomes at 2 and 5 years following randomisation to early reconstruction plus exercise therapy versus exercise therapy with optional delayed ACL reconstruction.^6 7^ Irrespective of treatment strategy, a proportion of individuals experience persistent knee difficulties and unsatisfactory outcomes following ACL rupture.^2 4 8–13^

Multiple studies have identified prognostic factors for poor postoperative outcomes following ACL reconstruction. Meniscal injury, concomitant meniscal surgery and full-thickness cartilage damage at the time of ACL reconstruction have been associated with worse outcomes (pain, symptoms, function, activity levels and QOL) up to 16 years after surgery.^14–21^ Psychological factors such as external locus of control, fear of reinjury and reduced knee self-efficacy have also been associated with poor functional and patient-reported outcomes after ACL reconstruction.^22–28^ Additionally, smoking and low education levels have predicted worse ACL reconstruction outcomes.^17 29^

Factors associated with an unsatisfactory long-term outcome following ACL rupture may differ in ACL-reconstructed and non-operatively managed persons. Better understanding of such differences could support individualised treatment choices to optimise outcomes following ACL rupture. Using data collected in the KANON trial,^6 7 30^ the objectives of the present study were to identify injury-related, patient-reported and treatment-related prognostic factors for 5-year patient-reported outcomes in ACL-ruptured individuals and to compare prognostic factors between the three as-treated groups (early reconstruction plus exercise therapy, exercise therapy plus delayed reconstruction and exercise therapy alone).

## Methods

### Study design and participants

This is an exploratory ‘as-treated’ analysis from the KANON trial (Current Controlled Trials ISRCTN84752559), a prospective RCT on surgical versus non-surgical treatment strategies of acute ACL rupture. The KANON trial enrolled active adults aged 18–35 years who presented to the Departments of Orthopaedics at Skåne University Hospital, Lund and Helsingborg Hospital, Sweden with an acute ACL rupture (injured within the preceding 4 weeks). Major exclusion criteria were professional athletes, less than moderately active individuals, previous knee injury, total collateral ligament rupture, full-thickness cartilage lesion visualised on MRI and extensive meniscal fixation. We have described details of recruitment process, inclusion and exclusion criteria and randomisation.^6 7 30^

The objective of the KANON trial was to compare outcomes between individuals randomised to a strategy of early ACL reconstruction plus supervised exercise therapy (n=62) and supervised exercise therapy with the option of having a delayed ACL reconstruction if needed (n=59). Twenty-three (39%) of the latter group had a delayed ACL reconstruction within the first 2 years^7^ and an additional 7 had a delayed reconstruction using a similar procedure in the following 3 years.^6^ One participant from the early reconstruction plus exercise therapy group was lost to follow-up between baseline and 5 years. Two patients assigned to early reconstruction plus exercise therapy did not have ACL reconstruction or attended <10 rehabilitation visits and were excluded from the as-treated analysis, presenting 59, 30 and 29 individuals in the early reconstruction plus exercise therapy, exercise therapy plus delayed reconstruction and exercise therapy alone groups at 5 years.^6^ Between baseline and 5-year follow-up, two participants received a revision ACL reconstruction, two individuals ruptured their ACL graft but did not undergo revision surgery and five underwent an ACL reconstruction in their contralateral knee (seven participants in the early reconstruction plus exercise therapy group and two participants in the exercise therapy plus delayed reconstruction group). Demographic data, patient-reported outcomes, imaging, biomarkers, strength and functional measures were collected at baseline, 3 months, 6 months, 12 months, 24 months and 5-year follow-up.

### Treatment strategy

Single-bundle autograft ACL reconstructions were performed using the patellar tendon (40 procedures) or hamstring tendon (51 procedures) in line with the preference of four senior surgeons who performed all procedures. All early ACL reconstructions were performed 46 weeks following randomisation. Timing of delayed ACL reconstructions ranged from 2 to 56 months after randomisation.^6^ Meniscal tears were treated by partial resection (and in a few cases partial fixation) when indicated by MRI and/or clinical findings, either at the time of ACL reconstruction or separately.^7^ Other surgical interventions were done as clinically indicated.^7^ Participants from all groups performed the same goal oriented, physiotherapist supervised neuromuscular exercise therapy programme at nine outpatient clinics.^7^

### Five-year outcomes

The Knee Injury and Osteoarthritis Outcome Score (KOOS) is valid for use in ACL-ruptured individuals and has a high test–retest reliability in patients with knee injury.^31^ The KOOS comprises five subscales, scored from 0 (worst) to 100 (best). The subscale addressing function in activities of daily living (ADL) is the least applicable to young, active individuals 5 years following ACL rupture, where near to normal scores are commonly reported irrespective of impairments in the other four subscales.^2 10^ Therefore, we excluded the ADL subscale, but included the other four KOOS subscales (pain, symptoms, sport and recreation function (sport/rec), QOL) as outcomes.

### Prognostic factors

#### Injury-related factors

Baseline cartilage and meniscus damage, osteochondral lesions and knee extension deficit were identified as injury-related factors with potential to impact 5-year outcome.

Baseline 1.5 T MRI examinations were used to grade concomitant baseline injuries using the Anterior Cruciate Ligament OsteoArthritis Score (ACLOAS)^32^ by a musculoskeletal radiologist with 11 years experience in standardised semi-quantitative MRI assessment of knee pathology and experience with using the ACLOAS (FWR). The ACLOAS is a whole joint scoring system devised for use in acutely ACL-injured knees covering acute osteochondral and other damage as well as pre-existing pathology commonly considered to be part of the osteoarthritis spectrum.^32^ All features were dichotomised into presence and absence for the purpose of this study (as described below), where the highest grade on each scale was taken for each participant. A full description of the ACLOAS scoring criteria has been published.^32^

The intraobserver reliability of the ACLOAS instrument including longitudinal assessment ranged between 0.52 (baseline, Hoffa synovitis) and 1.00 (several features), per cent agreement between 52% (all time points, Hoffa synovitis) and 100% (several features). Interobserver reliability ranged between 0.00 and 1.00, which is explained by low frequency of some of the features. Altogether, 73% of all assessed 142 parameters showed weighted-kappa values between 0.80 and 1.00% and 92% showed agreement above 80%.^32^

Cartilage damage was assessed using all available sequences and graded based on percentage of affected area and depth of damage in any given subregion. For this study, a cartilage defect was defined as a score of 2 (focal partial thickness defect affecting ≤10% of subregional area) or above.^32^

Baseline osteochondral surface damage was assessed in two dimensions, type of injury and size. For this study, an osteochondral lesion was defined as a score of ≥1 (subchondral fracture, osteochondral depression or detached fracture).^32^ Using these criteria, subchondral traumatic bone marrow lesions without a fracture and/or without articular surface damage were not considered osteochondral lesions.

Meniscal damage was assessed using sagittal sequences (for the anterior and posterior horn regions) and coronal sequences (for the meniscal body). For this study, meniscal damage was defined as a score of ≥2 (including all meniscal tears and extrusions, not including intrameniscal hyperintensity not extending to meniscal surface).^32^

A knee extension deficit >10 degrees at baseline was also identified as an injury-related factor with potential to impact 5-year outcome.

#### Treatment-related factors

The total number of knee surgery events *not* involving ACL reconstruction/revision (ie, any surgery *not* performed in the same surgical session as early or delayed ACL reconstruction or revision) performed between baseline and 5-year follow-up will be described as ‘non-ACL surgery’ (categorised into none, one or two or more ‘non-ACL surgeries’). Experiencing ACL graft rupture or contralateral ACL rupture, the number of rehabilitation visits attended, and receiving early reconstruction plus exercise therapy, exercise therapy plus delayed reconstruction or exercise therapy alone were also identified as treatment-related factors with a potential to impact 5-year outcome.

#### Patient-reported factors

Baseline Medical Outcomes Study 36-item Short-Form health survey (SF-36) Mental Component Scores (MCS)^33^ and baseline KOOS_4_ scores (a mean value from pain, symptoms, sport/rec and QOL subscales) were included as patient-reported outcomes with potential to impact 5-year outcome. The Physical Component Score from the SF-36 was not included in analyses due to potential collinearity with the KOOS_4_ and injury-related variables.

#### Confounders

Sex, age, body mass index (BMI), preinjury activity (assessed using the Tegner activity score),^7^ education (university vs other) and smoking (never vs other) were identified as potential confounders and included in the adjusted analyses.

### Statistical analysis

Prognostic factors and confounders were selected using clinical reasoning and literature review. Selected variables were portrayed in direct acyclic graphs to minimise overadjustment bias and collider stratification bias, with reference to a six-step process towards unbiased estimates.^34^ Linear regression analysis was used to investigate prognostic factors for 5-year outcome. Since this was an exploratory analysis, we made no adjustment for multiple comparisons. However, making multiple comparisons with no adjustments increases risk of chance findings. All underlying assumptions were assessed prior to analyses (including normality, multicollinearity between prognostic factors and normality, linearity and homoscedasticity of residuals). Since the preinjury Tegner score was negatively skewed and violated several assumptions, it was dichotomised using a cut-off of 5–7 (lower-level recreational sport and activities) versus 8–9 (higher-level pivoting, contact sport). Variables with three categories were transformed to three binary variables for use in multivariable analyses and the variable with the greatest sample size was used as a reference group. Adjusted effect estimates are reported for the entire cohort and within treatment groups in terms of regression coefficients and the estimation uncertainty is presented using 95% CI. Crude effect estimates are presented in an online supplementary appendix. Data were incomplete for three variables (smoking n=11 missing (9%), BMI n=2 missing (2%), SF-36 MCS n=1 missing (1%)). All missing data were assumed to be missing at random and no important differences were observed between individuals with complete and incomplete data.^35^ Multiple imputation using 40 iterations was performed to account for missing values using the Markov Chain Monte Carlo technique.^35^ Consistency between imputation iterations and convergence between complete data and imputed data was assessed by comparing proportions, descriptors and regression coefficients. All analyses and multiple imputation was performed using Stata/IC V.14.1 following consultation with a statistician (JR).

10.1136/bjsports-2016-097124.supp1 Supplementary data

## Results

### Participant characteristics

Participant characteristics including demographics, prognostic factors and 5-year KOOS outcomes for each treatment group are described in table 1.

**Table 1:** Participant characteristics

	All participants (n=118)	Early reconstruction plus exercise therapy (n=59)	Exercise therapy plus delayed reconstruction (n=30)	Exercise therapy alone (n=29)
Demographics				
Sex, male (n (%))	86 (73)	47 (80)	19 (63)	20 (69)
Age at baseline (mean (SD))	26 (5)	27 (5)	25 (5)	26 (5)
Current or past smoker at baseline (n (%))	30 (28)	17 (32)	5 (19)	8 (31)
Education, university level (n (%))	44 (37)	21 (36)	13 (43)	10 (35)
Preinjury Tegner activity level 8 or 9 (n (%))	79 (67)	39 (66)	22 (73)	18 (62)
Body mass index (mean (SD))	24 (3)	24 (3)	23 (2)	24 (3)
Prognostic factors				
Cartilage defect at baseline (n (%))	31 (26)	18 (31)	5 (17)	8 (28)
Meniscus damage at baseline (n (%))	30 (25)	18 (31)	5 (17)	7 (24)
Osteochondral lesion at baseline (n (%))	77 (65)	40 (68)	22 (73)	15 (52)
Extension deficit at baseline (n (%))	67 (57)	30 (51)	20 (67)	17 (59)
Graft rupture or contralateral ACL rupture (n (%))	9 (8)	7 (12)	2 (7)	0 (0)
Non-ACL surgery occasions (n (%))				
*None*	64 (54)	37 (63)	14 (47)	13 (45)
*One*	38 (32)	17 (29)	12 (40)	9 (31)
*Two or more*	16 (14)	5 (9)	4 (13)	7 (24)
SF-36 MCS at baseline (mean (SD))	67 (19)	68 (20)	65 (20)	66 (17)
KOOS_4_ at baseline (mean (SD))	37 (14)	37 (16)	36 (11)	38 (13)
Rehabilitation visits (mean (SD))	59 (36)	65 (36)	70 (37)	36 (23)
Five-year outcomes				
KOOS-pain (mean (SD))	91 (12)	91 (12)	91 (13)	92 (12)
KOOS-symptoms (mean (SD))	84 (16)	82 (17)	85 (14)	89 (16)
KOOS-sport/rec (mean (SD))	77 (23)	76 (23)	78 (26)	81 (22)
KOOS-QOL (mean (SD))	70 (22)	71 (21)	72 (24)	66 (23)

Due to missing values, sample size was reduced for the following: smoking n=107, BMI n=116, SF-36 MCS n=117. Education: university degree versus all lower levels of education. Pre-injury Tegner activity level: levels 8 or 9 (higher-level pivoting, contact sport) as opposed to 5–7 (lower-level recreational sport and activities). Graft rupture or contralateral ACL rupture: 4 were ACL graft ruptures (2 received revision ACL reconstruction) and 5 were contralateral ACL ruptures (all received contralateral ACL reconstruction); non-ACL surgery: total number of knee surgery events *not* performed in the same surgical session as ACL reconstruction or revision.

ACLR, ACL reconstruction; KOOS_4_, a mean score from four Knee injury and Osteoarthritis Outcome Score subscales (pain, symptoms, sport/rec and QOL) KOOS and SF-36 scores range from 0 (worst) to 100 (best); MCS, Mental Component Score; QOL, quality of life; sport/rec, sport and recreation function; SF-36, 36-item Short-Form.

#### All participants (n=118)

After adjustment for potential confounders, experiencing an ACL graft rupture or contralateral ACL rupture were prognostic factors for more knee pain and symptoms, worse sport and recreation function and reduced QOL (table 2). Having at least one knee surgery  not involving ACL reconstruction or revision, was also predictive of worse 5-year patient-reported outcomes. Reporting a 5-point worse SF-36 MCS at baseline predicted an estimated 1-point worse KOOS-pain score at follow-up. Individuals managed with exercise therapy alone reported an estimated 10-point *better* KOOS-symptoms score at 5 years, compared with people who underwent an early reconstruction plus exercise therapy (table 2).

**Table 2:** A multivariable adjusted model exploring prognostic factors for patient-reported outcomes at 5 years for all participants (n=118)

	KOOS-pain			KOOS-symptoms			KOOS-sport/rec			KOOS-QOL		
	Effect	95% CI	p Value	Effect	95% CI	p Value	Effect	95% CI	p Value	Effect	95% CI	p Value
Baseline cartilage defect	−1.3	−6.8 to 4.1	0.63	−5.3	−12.5 to 1.9	0.15	−4.9	−15.8 to 6.1	0.38	−0.5	−10.9 to 9.9	0.92
Baseline meniscus damage	−1.1	−6.6 to 4.4	0.69	−1.5	−8.8 to 5.7	0.68	−2.4	−13.5 to 8.6	0.66	−2.7	−13.2 to 7.8	0.61
Baseline osteochondral lesion	−0.8	−5.6 to 3.9	0.73	1.4	−4.9 to 7.7	0.66	−3.4	−13.0 to 6.2	0.48	−3.9	−13.0 to 5.2	0.40
Baseline extension deficit	−2.4	−7.1 to 2.4	0.33	−1.2	−7.5 to 5.1	0.71	−4.1	−13.6 to 5.4	0.40	−4.5	−13.6 to 4.5	0.32
Graft/ contralateral ACL rupture	**−12.0**	**−20.2 to −3.7**	**0.01**	−7.5	−18.4 to 3.3	0.17	**−24.1**	**−40.5 to −7.6**	**0.01**	**−23.6**	**−39.2 to −7.9**	**0.003**
1 non-ACL surgery*	**−5.7**	**−10.5 to −0.9**	**0.02**	**−9.5**	**−15.8 to −3.2**	**0.004**	**−9.7**	**−19.3 to −0.1**	**0.05**	−5.8	−14.9 to 3.3	0.21
≥2 non-ACL surgeries*	−7.0	−14.6 to 0.5	0.07	**−14.5**	**−24.4 to −4.6**	**0.01**	−13.6	−28.7 to 1.4	0.08	**−16.9**	**−31.2 to −2.6**	**0.02**
SF-36 MCS at baseline	**0.2**	**0.0 to 0.3**	**0.01**	0.1	−0.1 to 0.3	0.22	0.1	−0.1 to 0.3	0.41	0.1	−0.1 to 0.4	0.26
KOOS_4_ at baseline	0.0	−0.2 to 0.2	0.82	0.2	0.0 to 0.5	0.07	0.2	−0.2 to 0.5	0.33	0.2	−0.1 to 0.5	0.27
Rehabilitation visits	0.0	−0.1 to 0.1	0.74	0.0	−0.1 to 0.1	0.52	0.1	0.0 to 0.3	0.12	0.1	−0.1 to 0.2	0.27
Delayed ACLR†	−0.3	−5.6 to 5.1	0.92	2.5	−4.5 to 9.5	0.48	0.9	−9.7 to 11.6	0.86	−1.9	−9.2 to 11.0	0.74
Exercise therapy alone†	1.4	−4.5 to 7.4	0.63	**10.1**	**2.3 to 17.9**	**0.01**	9.1	−2.8 to 20.9	0.13	0.9	−13.1 to 9.4	0.86

*No non-ACL surgeries were used as a reference category in multivariable analysis.

†Early ACL reconstruction was used as a reference category in multivariable analysis.

Effect (regression coefficient): the estimate of the average change in a KOOS subscale (scores range from 0 (worst) to 100 (best)) that corresponds to a 1-unit change in the prognostic factor (1-unit=1 point on a 0–100 scale for KOOS_4_ and SF-36 MCS). All estimates are adjusted for sex, age, smoking, education level and preinjury activity level. Crude unadjusted estimates are presented in see online supplementary appendix. Non-ACL surgery: total number of knee surgery events *not* performed in the same surgical session as ACL reconstruction or revision. KOOS and SF-36 scores range from 0 (worst) to 100 (best).

Statistically significant effects (p<0.05) are highlighted in bold.

ACLR, ACL reconstruction; KOOS_4_, a mean score from four Knee injury and Osteoarthritis Outcome Score subscales (pain, symptoms, sport/rec and QOL); MCS, Mental Component Score; SF-36, 36-item Short-Form.

#### Early reconstruction plus exercise therapy (n=59)

Participants who received an early reconstruction plus exercise therapy and had baseline meniscus damage reported an estimated 14-point worse KOOS-sport/rec score at follow-up compared with early reconstructed people without baseline meniscus damage (table 3). Participants who underwent an early reconstruction with baseline osteochondral injury reported an estimated 12-point worse KOOS-QOL score at 5 years compared with early reconstructed individuals without osteochondral injury. Undergoing one or more non-ACL surgery following early ACL reconstruction was a prognostic factor for worse patient-reported outcomes at 5 years (table 3). Worse baseline KOOS_4_ scores were associated with worse knee symptoms, reduced sport and recreation function and decreased QOL at 5 years.

**Table 3:** A multivariable adjusted model exploring prognostic factors for patient-reported outcomes at 5 years for the early reconstruction plus exercise therapy group

	KOOS-pain			KOOS-symptoms			KOOS-sport/rec			KOOS-QOL		
	Effect	95% CI	p Value	Effect	95% CI	p Value	Effect	95% CI	p Value	Effect	95% CI	p Value
Baseline cartilage defect	2.5	−4.7 to 9.7	0.49	−3.8	−14.5 to 6.9	0.48	−3.0	−17.4 to 11.3	0.67	9.0	−4.4 to 22.3	0.18
Baseline meniscus damage	−4.4	−11.0 to 2.2	0.19	−6.2	−16.0 to 3.6	0.21	**−14.4**	**−27.6 to −1.3**	**0.03**	−11.9	−24.2 to 0.3	0.06
Baseline osteochondral lesion	−5.4	−11.7 to 1.0	0.10	0.4	−9.2 to 9.9	0.94	−3.7	−16.5 to 9.1	0.56	**−12.3**	**−24.2 to −0.4**	**0.04**
Baseline extension deficit	−3.3	−9.4 to 2.7	0.27	1.3	−7.8 to 10.4	0.77	−5.3	−17.5 to 6.9	0.38	−6.1	−17.4 to 5.3	0.29
Graft/contralateral ACL rupture	−0.5	−9.8 to 8.7	0.91	2.6	−11.1 to 16.3	0.70	−2.1	−20.5 to 16.4	0.82	−2.3	−19.5 to 14.8	0.79
1 non-ACL surgery*	**−8.0**	**−14.4 to −1.5**	**0.02**	**−11.2**	**−20.9 to −1.6**	**0.02**	**−15.1**	**−28.0 to −2.1**	**0.02**	−9.8	−21.8 to 2.2	0.11
≥2 non-ACL surgeries*	**−16.9**	**−27.7 to −6.0**	**0.003**	**−24.0**	**−40.2 to −7.8**	**0.01**	**−25.8**	**−47.6 to −4.0**	**0.02**	**−20.2**	**−40.4 to 0.1**	**0.05**
SF-36 MCS at baseline	0.1	−0.1 to 0.2	0.42	−0.1	−0.3 to 0.2	0.67	−0.1	−0.4 to 0.3	0.63	−0.1	−0.4 to 0.2	0.59
KOOS_4_ baseline	0.2	0.0 to 0.4	0.07	**0.5**	**0.1 to 0.8**	**0.01**	**0.5**	**0.0 to 0.9**	**0.04**	**0.7**	**0.3 to 1.1**	**0.002**
Rehabilitation visits	0.0	0.0 to 0.1	0.31	0.1	−0.1 to 0.2	0.44	0.2	0.0 to 0.4	0.07	0.1	−0.1 to 0.3	0.27

*No non-ACL surgeries was used as a reference category in multivariable analysis.

Effect (regression coefficient): the estimate of the average change in a KOOS subscale (scores range from 0 (worst) to 100 (best)) that corresponds to a 1-unit change in the prognostic factor (1-unit=1 point on a 0–100 scale for KOOS_4_ and SF-36 MCS). All estimates are adjusted for sex, age, smoking, education level and preinjury activity level. Crude unadjusted estimates are presented in see online supplementary appendix. Non-ACL surgery: total number of knee surgery events *not* involving ACL reconstruction or revision. KOOS and SF-36 scores range from 0 (worst) to 100 (best).

Statistically significant effects (p<0.05) are highlighted in bold.

KOOS_4_, a mean score from four Knee injury and Osteoarthritis Outcome Score subscales (pain, symptoms, sport/rec and QOL); MCS, Mental Component Score; SF-36, 36-item Short-Form.

#### Exercise therapy plus delayed reconstruction (n=30)

In the exercise therapy plus delayed reconstruction group, baseline meniscus damage predicted an estimated 14-point *better* KOOS-pain score at follow-up compared with no baseline meniscal injury (table 4). Experiencing ACL graft rupture or contralateral ACL rupture after a delayed reconstruction was a prognostic factor for more knee pain, worse sport/recreation function and reduced QOL at 5 years, although only two individuals in this group experienced a contralateral ACL or graft rupture.

**Table 4:** A multivariable adjusted model exploring prognostic factors for patient-reported outcomes at 5 years for the exercise therapy plus delayed reconstruction group

	KOOS-pain			KOOS-symptoms			KOOS-sport/rec			KOOS-QOL		
	Effect	95% CI	p Value	Effect	95% CI	p Value	Effect	95% CI	p Value	Effect	95% CI	p Value
Baseline cartilage defect	2.7	−11.2 to 16.5	0.68	0.0	−23.1 to 23.1	1.00	12.7	−19.8 to 45.3	0.41	8.9	−19.1 to 36.8	0.50
Baseline meniscus damage	**14.3**	**0.7 to 27.9**	**0.04**	4.4	−18.4 to 27.2	0.68	27.4	−3.8 to 58.7	0.08	22.8	−5.0 to 50.5	0.10
Baseline osteochondral lesion	6.2	−5.8 to 18.2	0.28	3.2	−16.7 to 23.2	0.73	2.9	−25.1 to 31.0	0.82	1.3	−23.0 to 25.5	0.91
Baseline extension deficit	−1.4	−13.2 to 10.3	0.79	2.9	−16.8 to 22.6	0.76	4.5	−22.5 to 31.5	0.72	−1.2	−25.2 to 22.9	0.92
Graft/contralateral ACL rupture	**−33.7**	**−52.5 to −14.9**	**0.002**	−29.1	−60.6 to 2.4	0.07	**−71.0**	**−114.3 to −27.7**	**0.004**	**−67.4**	**−105.7 to −29.2**	**0.002**
1 non-ACL surgery*	−4.3	−16.3 to 7.8	0.45	−5.4	−25.5 to 14.7	0.57	−14.2	−42.3 to 13.9	0.29	−7.1	−31.5 to 17.4	0.54
≥2 non-ACL surgeries*	6.2	−10.1 to 22.5	0.42	6.2	−20.9 to 33.2	0.63	0.2	−40.0 to 40.4	0.99	7.6	−24.8 to 40.0	0.62
SF-36 MCS at baseline	−0.2	−0.4 to 0.1	0.17	−0.2	−0.7 to 0.2	0.23	−0.2	−0.8 to 0.4	0.56	−0.4	−0.9 to 0.2	0.16
KOOS_4_ baseline	−0.2	−0.7 to 0.3	0.35	0.1	−0.6 to 0.9	0.70	−0.3	−1.3 to 0.8	0.60	−0.4	−1.3 to 0.6	0.39
Rehabilitation visits	−0.1	−0.3 to 0.0	0.12	−0.1	−0.3 to 0.2	0.48	−0.1	−0.4 to 0.3	0.74	−0.1	−0.4 to 0.2	0.53

*No non-ACL surgeries was used as a reference category in multivariable analysis.

Effect (regression coefficient): the estimate of the average change in a KOOS subscale (scores range from 0 (worst) to 100 (best)) that corresponds to a 1-unit change in the prognostic factor (1-unit=1 point on a 0–100 scale for KOOS_4_ and SF-36 MCS). All estimates are adjusted for sex, age, smoking, education level and preinjury activity level. Crude unadjusted estimates are presented in see online supplementary appendix. Non-ACL surgery: total number of knee surgery events *not* performed in the same surgical session as ACL reconstruction or revision. KOOS and SF-36 scores range from 0 (worst) to 100 (best).

Statistically significant effects (p<0.05) are highlighted in bold.

KOOS_4_, a mean score from four Knee injury and Osteoarthritis Outcome Score subscales (pain, symptoms, sport/rec and QOL); MCS, Mental Component Score; SF-36, 36-item Short-Form.

#### Exercise therapy alone (n=29)

Participants managed with exercise therapy alone who received one non-ACL surgery reported an estimated 14-point worse KOOS-pain score at 5 years compared with people remaining surgery free (table 5).

**Table 5:** A multivariable adjusted model exploring prognostic factors for patient-reported outcomes at 5 years for the exercise therapy alone group

	KOOS-pain			KOOS-symptoms			KOOS-sport/rec			KOOS-QOL		
	Effect	95% CI	p Value	Effect	95% CI	p Value	Effect	95% CI	p Value	Effect	95% CI	p Value
Baseline cartilage defect	−11.9	−30.4 to 6.6	0.18	−22.3	−46.2 to 1.5	0.06	−20.2	−58.5 to 18.0	0.27	−10.3	−54.3 to 33.7	0.61
Baseline meniscus damage	9.4	−10.2 to 29.0	0.31	11.1	−12.9 to 35.1	0.33	28.8	−12.8 to 70.5	0.16	−3.1	−46.3 to 40.2	0.88
Baseline osteochondral lesion	−3.5	−16.5 to 9.5	0.56	−1.4	−17.3 to 14.5	0.85	−13.9	−41.4 to 13.7	0.29	−2.0	−31.2 to 27.3	0.89
Baseline extension deficit	7.2	−12.3 to 26.6	0.44	0.7	−23.4 to 24.8	0.95	15.0	−25.6 to 55.6	0.43	2.8	−40.2 to 45.9	0.89
Graft/contralateral ACL rupture	–	–	–	–	–	–	–	–	–	–	–	–
1 non-ACL surgery*	**−14.0**	**−27.9 to −0.1**	**0.049**	−13.8	−30.8 to 3.3	0.10	−22.8	−52.2 to 6.6	0.12	−10.4	−41.2 to 20.5	0.48
≥2 non-ACL surgeries*	−6.5	−32.4 to 19.5	0.59	−18.2	−50.1 to 13.7	0.24	−18.6	−73.2 to 36.1	0.47	−1.3	−59.6 to 56.9	0.96
SF-36 MCS at baseline	0.2	−0.2 to 0.6	0.30	0.2	−0.3 to 0.8	0.30	0.3	−0.5 to 1.2	0.40	0.5	−0.4 to 1.5	0.23
KOOS_4_ baseline	0.3	−0.3 to 0.9	0.30	0.3	−0.4 to 1.1	0.38	0.6	−0.6 to 1.9	0.28	0.1	−1.2 to 1.5	0.82
Rehabilitation visits	−0.1	−0.6 to 0.3	0.47	0.0	−0.5 to 0.6	0.84	−0.4	−1.2 to 0.5	0.39	−0.5	−1.4 to 0.4	0.22

*No non-ACL surgeries was used as a reference category in multivariable analysis.

Effect (regression coefficient): the estimate of the average change in a KOOS subscale (scores range from 0 (worst) to 100 (best)) that corresponds to a 1-unit change in the prognostic factor (1-unit=1 point on a 0–100 scale for KOOS_4_ and SF-36 MCS). All estimates are adjusted for sex, age, smoking, education level and preinjury activity level. Crude unadjusted estimates are presented in see online supplementary appendix. Non-ACL surgery: total number of knee surgery events *not* performed in the same surgical session as ACL reconstruction or revision. KOOS and SF-36 scores range from 0 (worst) to 100 (best).

Statistically significant effects (p<0.05) are highlighted in bold.

KOOS_4_, a mean score from four Knee injury and Osteoarthritis Outcome Score subscales (pain, symptoms, sport/rec and QOL); MCS, Mental Component Score; SF-36, 36-item Short-Form.

## Results summary

An overview of prognostic factors for 5-year outcome is provided in table 6.

**Table 6:** A summary of prognostic factors for 5-year patient-reported outcomes

	All participants (n=118)	Early reconstruction plus exercise therapy (n=59)	Exercise therapy plus delayed reconstruction (n=30)	Exercise therapy alone (n=29)
Baseline meniscus damage	–	Worse sport/rec	Less pain	–
Osteochondral lesion	–	Worse QOL	–	–
Graft rupture/ contralateral ACL rupture	More pain Worse sport/rec Worse QOL	–	More pain Worse sport/rec Worse QOL	–
1 non-ACL surgery*	More pain More symptoms Worse sport/rec	More pain More symptoms Worse sport/rec	–	More pain
≥2 non-ACL surgeries*	More symptoms Worse QOL	More pain More symptoms Worse sport/rec Worse QOL	–	–
Worse SF-36 MCS	More pain	–	–	–
Worse baseline KOOS_4_ scores	–	More symptoms Worse sport/rec Worse QOL	–	–
Exercise therapy alone†	Less symptoms	–	–	–

*Compared with no non-ACL surgeries.

†Compared with the early reconstruction plus exercise therapy group.

Statistically significant effects (p<0.05) are presented for prognostic factors associated with 5-year outcome(s) assessed using adjusted multivariable analyses.

‘Worse’ SF-36 MCS and KOOS_4_ scores represent lower scores on these measures which range from 100 (best possible score) to 0 (worse possible score).

Non-ACL surgery: total number of knee surgery events not performed in the same surgical session as ACL reconstruction or revision.

KOOS_4_, a mean score from four Knee injury and Osteoarthritis Outcome Score subscales (pain, symptoms, sport/rec and QOL); MCS, Mental Component Score; QOL, quality of life; sport/rec, sport and recreation function; SF-36, 36-item Short-Form.

## Discussion

This is the first study to explore prognostic factors for a cohort comprising young active individuals with an acutely ruptured ACL managed with early reconstruction plus exercise therapy, exercise therapy plus delayed reconstruction or exercise therapy alone. When all participants were analysed together, graft rupture/contralateral ACL rupture, non-ACL surgery, worse baseline SF-36 MCS and undergoing early reconstruction plus exercise therapy compared with exercise therapy alone were prognostic factors for worse 5-year outcomes on one or more KOOS subscales. Baseline cartilage injury, baseline extension deficit and number of rehabilitation visits were not related to 5-year outcomes for all participants or any of the treatment groups. A further exploratory analysis of treatment groups revealed differences in prognostic factors, suggesting that delaying ACL reconstruction and managing ACL rupture with exercise therapy alone may alter prognostic factors for 5-year outcomes in a positive direction. Our findings suggest that young, active individuals with acute ACL rupture who have concomitant meniscus injury, and those reporting more severe knee pain, symptoms and impaired function in the early phase of injury, may benefit most from commencing exercise therapy before considering ACL reconstruction.

### Baseline meniscal damage and osteochondral lesions

For the early reconstruction plus exercise therapy group, osteochondral injury was a prognostic factor for worse QOL and meniscus damage was related to worse sport and recreation function at 5 years. In contrast, baseline meniscus damage was a prognostic factor for *less* pain at follow-up for the exercise therapy plus delayed reconstruction group. The mechanisms behind this surprising finding are not clear, but sustaining a second knee insult in the form of an early ACL reconstruction shortly after a previous knee trauma may increase the likelihood of experiencing persistent postoperative difficulties. The additional sequelae of ACL reconstruction, including surgical trauma to intra-articular structures and a period of prolonged joint inflammation and altered weight bearing, may provide a suboptimal environment for healing of meniscus and other joint tissues, compared with initial management with a goal-oriented exercise therapy programme guided by knee pain and symptoms.

People with baseline meniscus injury who underwent a delayed reconstruction may have experienced more pain related to mechanical knee issues (such as instability, persistent swelling or limited range of motion) compared with those without meniscus injury. Reconstructive surgery may have been more successful in relieving pain in this group, compared with those electing to undergo surgery for a range of other reasons, including a desire to gain preinjury status, a pre-existing preference for surgery and finding exercise therapy boring and time consuming.^36^

These explanations are speculative and further research is needed to explore the relationship between management strategies for ACL rupture, meniscus damage and long-term outcomes.

### Non-ACL surgery

As many as one in five ACL-reconstructed individuals undergo additional subsequent surgery to the index knee within 6 years of ACL reconstruction.^37^ Subsequent surgery was found to predict more knee pain at 2 and 6 years after ACL reconstruction^13^ and worse QOL in people with knee difficulties 5–20 years after ACL reconstruction.^38^ In the present study, undergoing non-ACL surgery was related to worse patient-reported outcomes for all participants, especially for the early reconstruction plus exercise therapy group. The reason for worse outcomes in those who underwent non-ACL surgery after ACL rupture is unknown. However, it is likely that knee pain, symptoms, activity limitations and impaired QOL are common drivers of non-ACL surgery, while it is uncertain if these symptoms are relieved by the surgery. Further investigation is required to assess the effectiveness of specific surgical procedures in improving long-term outcomes for people with knee difficulties after ACL reconstruction. Including a range of preoperative and postoperative patient-reported measures and reporting the patient’s and surgeon’s rationale for surgery may provide new insights.

### ACL graft rupture or contralateral ACL rupture

Our finding of worse 5-year outcomes in people who experience an ACL graft rupture or contralateral rupture is in line with previous research.^39–42^ Of concern is that as many as one in four individuals suffer a graft rerupture or contralateral ACL rupture within 15 years of ACL reconstruction^43^ and this is most common in adolescents who undergo ACL reconstruction.^44^ The possibility of sustaining an ACL graft rupture that increases the risk of poor long-term outcomes should be discussed with patients weighing up ACL management options.

### Baseline KOOS_4_ and SF-36 MCS scores

Worse baseline KOOS_4_ score was a prognostic factor for worse 5-year outcomes following early reconstruction plus exercise therapy and impaired baseline SF-36 MCS predicted more knee pain at follow-up for all participants. Individuals who report worse baseline KOOS_4_ and SF-36 MCS may exhibit psychological traits that are associated with reporting worse outcomes after ACL reconstruction, such as reduced knee self-efficacy, pain catastrophising, pessimism, poor knee confidence and external locus of control.^22–24 45 46^ Low baseline KOOS_4_ scores also reflect more physical impairment and this may predispose an individual to worse postoperative outcomes. Individuals who report worse KOOS scores prior to reconstruction may benefit from postponing surgery and commencing exercise therapy before considering surgical reconstruction. This suggestion is consistent with a prior study showing that 5 weeks of intensive exercise therapy prior to surgery resulted in a better postoperative outcome.^47 48^ Baseline patient-reported and psychological measures may be used in future studies to advise individuals better suited to a specific ACL management strategy.

### Early reconstruction plus exercise therapy compared with exercise therapy alone

Early reconstruction within 10 weeks of injury plus exercise therapy was a prognostic factor for more knee symptoms at 5 years compared with management with exercise therapy alone. It is important to note that patients managed with exercise therapy alone in this study are not generalisable to all ACL-ruptured patients receiving exercise therapy, since those who went on to have a delayed ACL reconstruction were excluded from this group. Notably, reporting worse preoperative KOOS scores prior to early reconstruction plus exercise therapy was related to more knee symptoms at 5 years, but this was not the case for individuals treated with exercise therapy plus delayed reconstruction. These findings strengthen the possibility that commencing exercise therapy and enabling the acute signs of injury to subside prior to considering ACL reconstruction may benefit long-term outcomes. Surgical reconstruction of the ACL causes iatrogenic damage to knee structures, which may increase the likelihood of experiencing future knee symptoms compared with management with exercise therapy alone. Components of ACL reconstruction including surgical incision, graft harvesting and bone drilling may contribute to long-term complaints including numbness and altered sensation, kneeling difficulties and patellofemoral pain.^12 49 50^

### Limitations and strengths

This was a post hoc exploratory, hypothesis-generating analysis of outcomes of an RCT on ACL treatment. Due to the exploratory nature of this analysis, we did not adjust for multiple comparisons. Furthermore, our subgroup analysis resulted in a reduced sample size, which may have increased the risk of spurious results and reduced the likelihood of finding statistically significant prognostic factors, in particular for the exercise therapy alone and exercise therapy plus delayed reconstruction groups. This may also explain the wide 95% CIs suggesting uncertainty in some of the estimates. Considering these limitations, we emphasise that larger prospective cohort studies are needed before these findings are applied in the clinical setting.

Although most estimates exceeded previously reported minimally clinically important difference (MCID) values for the KOOS, we refrained from interpreting findings relative to MCID since the MCID is likely to be different for patients managed with reconstruction and those managed with exercise therapy alone. Furthermore, the patient characteristics in the KANON trial differ from the characteristics of patients where MCID values were derived, making it in appropriate for use in this study.

Our study is the first to explore prognostic factors for long-term outcome in different treatment groups in a high-quality RCT. Further strengths include the depth of baseline data available for use as prognostic factors, the low dropout rate over 5 years and the standardised, monitored goal-oriented rehabilitation regime that all participants undertook.

### Conclusion

This exploratory investigation suggests that young active adults with an acute ACL tear to a previously uninjured knee with baseline meniscus damage, an osteochondral lesion or more self-reported knee impairment, treated with early reconstruction plus exercise therapy, may experience worse 5-year outcomes compared with those with similar baseline characteristics treated with exercise therapy with or without a delayed reconstruction. This information may be useful in guiding further research and could assist to identify individuals who may benefit most from non-operative or surgical management of ACL rupture.

**What are the findings?**

- Delaying ACL reconstruction and managing ACL rupture with exercise therapy alone may shift prognostic factors for 5-year outcomes in a positive direction.
- Individuals reporting worse Knee Injury and Osteoarthritis Outcome Score symptoms, sport/recreation and QOL scores following acute ACL rupture may benefit from commencing exercise therapy before considering ACL reconstruction.
- Research is needed to determine whether individuals with baseline meniscus damage benefit from delaying ACL reconstruction and commencing exercise therapy.
- Further investigation is required to assess the effectiveness of specific surgical procedures in improving long-term outcomes for people with knee difficulties after ACL reconstruction.

**How might it impact on clinical practice in the future?**

If further research supports these findings:

- Patient characteristics including concomitant meniscus damage and a higher degree of self-reported knee impairment within 4 weeks of ACL rupture, may be used to identify individuals most likely to benefit from commencing exercise therapy before considering ACL reconstruction.
- To enable an individual with an acute ACL rupture to make an informed decision about management options, clinicians should provide a personalised discussion regarding the likelihood of better or worse outcomes with each management option, tailored to the individual’s personal and injury-related characteristics.
- Clinicians should assure that individuals with knee complaints after ACL rupture receive unbiased, evidence-based information on the benefits and harms associated with different management options.
