# Supplementary material for: Delaying ACL reconstruction and treating with exercise therapy alone may alter prognostic factors for 5-year outcome: an exploratory analysis of the KANON trial
Source: Br J Sports Med. 2017 May 17;51(22):1622–9. doi: 10.1136/bjsports-2016-097124 (PMC5754848; doi:10.1136/bjsports-2016-097124)
Supplement: Supplementary data [file bjsports-2016-097124supp001.docx]

|  | **KOOS-Pain** | | | **KOOS-Symptoms** | | | **KOOS-Sport-Rec** | | | **KOOS-QOL** | | |
| --- | --- | --- | --- | --- | --- | --- | --- | --- | --- | --- | --- | --- |
|  | Crude effect | 95% CI | p | Crude effect | 95% CI | p | Crude effect | 95% CI | p | Crude effect | 95% CI | p |
| Baseline cartilage defect | -3.5 | -8.4 to 1.4 | 0.16 | **-7.9** | **-14.4 to -1.3** | **0.02** | -8.4 | -18.0 to 1.2 | 0.09 | -4.7 | -13.9 to 4.5 | 0.31 |
| Baseline meniscus damage | -4.5 | -9.4 to 0.5 | 0.08 | **-8.0** | **-14.6 to -1.3** | **0.02** | -7.3 | -17.1 to 2.4 | 0.14 | -6.7 | -16.0 to 2.5 | 0.15 |
| Baseline osteochondral lesion | -3.4 | -7.9 to 1.1 | 0.14 | -3.1 | -9.3 to 3.1 | 0.32 | -8.2 | -17.1 to 0.7 | 0.07 | -5.4 | -13.9 to 3.1 | 0.21 |
| Baseline extension deficit | -3.3 | -7.6 to 1.1 | 0.14 | -3.6 | -9.5 to 2.3 | 0.23 | -5.5 | -14.1 to 3.1 | 0.21 | -6.1 | -14.2 to 2.0 | 0.14 |
| Graft/contralateral ACL rupture | **-9.3** | **-17.3 to -1.3** | **0.02** | -6.3 | -17.3 to 4.8 | 0.26 | **-21.9** | **-37.5 to -6.3** | **0.01** | **-19.2** | **-34.1 to -4.3** | **0.01** |
| No non-ACL surgery | **6.5** | **2.3 to 10.7** | **0.003** | **11.1** | **5.5 to 16.6** | **<0.001** | **10.0** | **1.6 to 18.4** | **0.02** | **9.8** | **1.9 to 17.8** | **0.02** |
| 1 non-ACL surgery | **-5.3** | **-9.8 to -0.7** | **0.02** | **-7.9** | **-14.0 to -1.7** | **0.01** | **-8.9** | **-17.9 to 0.1** | **0.05** | -4.5 | -13.1 to 4.2 | 0.31 |
| ≥2 non-ACL surgeries | -3.9 | -10.2 to 2.4 | 0.23 | **-8.8** | **-17.2 to -0.3** | **0.04** | -4.7 | -17.1 to 7.8 | 0.46 | **-12.5** | **-24.2 to -0.9** | **0.04** |
| SF-36 MCS at baseline | **0.2** | **0.1 to 0.3** | **0.002** | **0.2** | **0.0 to 0.3** | **0.05** | 0.1 | -0.1 to 0.4 | 0.23 | 0.2 | 0.0 to 0.4 | 0.09 |
| KOOS_4_ at baseline | **0.2** | **0.0 to 0.3** | **0.01** | **0.4** | **0.2 to 0.6** | **0.001** | **0.4** | **0.1 to 0.7** | **0.01** | **0.4** | **0.1 to 0.7** | **0.003** |
| Rehabilitation visits | 0.0 | -0.1 to 0.0 | 0.15 | **-0.1** | **-0.2 to 0.0** | **0.04** | 0.0 | -0.1 to 0.1 | 0.78 | 0.0 | -0.1 to 0.1 | 0.91 |
| Delayed ACLR | -0.2 | -5.2 to 4.8 | 0.93 | 0.2 | -6.6 to 7.0 | 0.96 | 0.1 | -9.8 to 9.9 | 0.99 | 2.5 | -6.8 to 11.8 | 0.60 |
| Exercise therapy alone | 1.0 | -4.0 to 6.0 | 0.70 | 6.5 | -0.2 to 13.2 | 0.06 | 5.2 | -4.7 to 15.1 | 0.30 | -4.5 | -13.9 to 4.8 | 0.34 |
| Early ACLR | -0.6 | -4.9 to 3.8 | 0.80 | -5.0 | -10.8 to 0.9 | 0.09 | -3.9 | -12.4 to 4.6 | 0.37 | 1.5 | -6.6 to 9.6 | 0.72 |

**Supplementary Appendix Table 1:** Crude (unadjusted) results for all participants (n=118)

Effect (regression coefficient): the estimate of the average change in a KOOS subscale (scores range from 0 (worst) to 100 (best)) that corresponds to a 1-unit change in the prognostic factor (1-unit = 1-point on a 0 to 100 scale for KOOS_4_ and SF-36 MCS)

Non-ACL surgery: total number of knee surgery events *not* performed in the same surgical session as ACL reconstruction or revision

KOOS and SF-36 scores range from 0 (worst) to 100 (best)

MCS: Mental Component Score

KOOS_4_: a mean score from four Knee injury and Osteoarthritis Outcome Score subscales (Pain, Symptoms, Sport/Rec and QOL)

ACLR: anterior cruciate ligament (ACL) reconstruction

**Statistically significant effects (p value <0.05) are highlighted in bold**

|  | **KOOS-Pain** | | | **KOOS-Symptoms** | | | **KOOS-Sport-Rec** | | | **KOOS-QOL** | | |
| --- | --- | --- | --- | --- | --- | --- | --- | --- | --- | --- | --- | --- |
|  | Crude effect | 95% CI | p | Crude effect | 95% CI | p | Crude effect | 95% CI | p | Crude effect | 95% CI | p |
| Baseline cartilage defect | -4.4 | -11.0 to 2.2 | 0.19 | **-11.1** | **-20.3 to -1.8** | **0.02** | **-16.3** | **-28.7 to -3.9** | **0.01** | -7.6 | -19.5 to 4.3 | 0.21 |
| Baseline meniscus damage | **-8.0** | **-14.3 to -1.6** | **0.02** | **-10.5** | **-19.8 to -1.2** | **0.03** | **-18.3** | **-30.5 to -6.1** | **0.004** | **-12.1** | **-23.8 to -0.4** | **0.04** |
| Baseline osteochondral lesion | **-6.8** | **-13.1 to -0.4** | **0.04** | -5.1 | -14.6 to 4.4 | 0.29 | -12.1 | -24.6 to 0.5 | 0.06 | **-16.3** | **-27.4 to -5.1** | **0.01** |
| Baseline extension deficit | -4.9 | -10.9 to 1.2 | 0.11 | -4.8 | -13.7 to 4.0 | 0.28 | -9.5 | -21.3 to 2.3 | 0.11 | -9.6 | -20.4 to 1.3 | 0.08 |
| Graft/contralateral ACL rupture | -2.5 | -12.1 to 7.0 | 0.60 | -0.3 | -14.2 to 13.5 | 0.97 | -7.1 | -25.7 to 11.5 | 0.45 | -9.1 | -26.2 to 7.9 | 0.29 |
| No non-ACL surgery | **10.8** | **5.1 to 16.5** | **<0.001** | **17.7** | **9.8 to 25.7** | **<0.001** | **21.1** | **9.9 to 32.3** | **<0.001** | **15.1** | **4.3 to 25.9** | **0.01** |
| 1 non-ACL surgery | **-6.7** | **-13.3 to -0.1** | **0.05** | **-11.8** | **-21.1 to -2.4** | **0.02** | **-16.0** | **-28.7 to -3.4** | **0.01** | -9.8 | -21.8 to 2.3 | 0.11 |
| ≥2 non-ACL surgeries | **-14.8** | **-25.1 to -4.4** | **0.01** | **-22.4** | **-37.3 to -7.4** | **0.004** | **-21.3** | **-42.3 to -0.4** | **0.05** | **-19.7** | **-39.0 to -0.4** | **0.05** |
| SF-36 MCS at baseline | **0.2** | **0.1 to 0.4** | **0.002** | 0.2 | -0.0 to 0.4 | 0.11 | 0.2 | -0.1 to 0.5 | 0.23 | **0.3** | **0.0 to 0.5** | **0.04** |
| KOOS_4_ at baseline | **0.3** | **0.1 to 0.5** | **0.001** | **0.5** | **0.3 to 0.8** | **<0.001** | **0.6** | **0.3 to 1.0** | **0.001** | **0.7** | **0.4 to 1.0** | **<0.001** |
| Rehabilitation visits | -0.1 | -0.2 to 0.0 | 0.13 | -0.1 | -0.22 to 0.0 | 0.10 | -0.1 | -0.2 to 0.1 | 0.55 | -0.1 | -0.2 to 0.1 | 0.35 |

**Supplementary Appendix Table 2:** Crude (unadjusted) results for participants treated with early ACL reconstruction (n=59)

Effect (regression coefficient): the estimate of the average change in a KOOS subscale (scores range from 0 (worst) to 100 (best)) that corresponds to a 1-unit change in the prognostic factor (1-unit = 1-point on a 0 to 100 scale for KOOS_4_ and SF-36 MCS)

Non-ACL surgery: total number of knee surgery events *not* performed in the same surgical session as ACL reconstruction or revision

KOOS and SF-36 scores range from 0 (worst) to 100 (best)

MCS: Mental Component Score

KOOS_4_: a mean score from four Knee injury and Osteoarthritis Outcome Score subscales (Pain, Symptoms, Sport/Rec and QOL)

ACLR: anterior cruciate ligament (ACL) reconstruction

**Statistically significant effects (p value <0.05) are highlighted in bold**

|  | **KOOS-Pain** | | | **KOOS-Symptoms** | | | **KOOS-Sport-Rec** | | | **KOOS-QOL** | | |
| --- | --- | --- | --- | --- | --- | --- | --- | --- | --- | --- | --- | --- |
|  | Crude effect | 95% CI | p | Crude effect | 95% CI | p | Crude effect | 95% CI | p | Crude effect | 95% CI | p |
| Baseline cartilage defect | -3.0 | -15.9 to 9.9 | 0.64 | -2.9 | -16.8 to 11.1 | 0.68 | 9.0 | -17.3 to 35.3 | 0.49 | 4.0 | -20.1 to 28.1 | 0.74 |
| Baseline meniscus damage | 9.0 | -3.5 to 21.5 | 0.15 | 4.0 | -9.9 to 17.9 | 0.56 | **24.6** | **-0.1 to 49.3** | **0.05** | 22.0 | -0.6 to 44.6 | 0.06 |
| Baseline osteochondral lesion | 6.0 | -4.7 to 16.7 | 0.26 | 6.3 | -5.3 to 17.9 | 0.27 | -1.9 | -22.3 to 22.3 | 1.00 | 5.0 | -15.2 to 25.3 | 0.61 |
| Baseline extension deficit | -1.3 | -11.5 to 9.0 | 0.80 | -0.2 | -11.3 to 10.9 | 0.97 | -1.5 | -22.4 to 19.4 | -0.15 | -1.3 | -20.4 to 17.9 | 0.89 |
| Graft/contralateral ACL rupture | **-31.8** | **-46.8 to -16.9** | **<0.001** | -17.9 | -37.6 to 1.9 | 0.08 | **-67.0** | **-96.8 to -37.1** | **<0.001** | **-60.0** | **-87.6 to -32.5** | **<0.001** |
| No non-ACL surgery | -2.5 | -12.2 to 7.1 | 0.60 | 1.8 | -8.7 to 12.2 | 0.73 | -12.7 | **-31.9 to 6.4** | **0.18** | -11.3 | -28.8 to 6.2 | 0.20 |
| 1 non-ACL surgery | -1.31 | -11.2 to 8.6 | 0.79 | -2.5 | -13.1 to 8.2 | 0.64 | **4.9** | **-15.2 to 24.9** | **0.50** | 4.7 | -13.6 to 23.0 | 0.60 |
| ≥2 non-ACL surgeries | 8.2 | -5.7 to 22.1 | 0.24 | 1.4 | -14.0 to 16.8 | 0.86 | **17.3** | **-10.9 to 45.6** | **0.22** | 14.7 | -11.2 to 40.5 | 0.26 |
| SF-36 MCS at baseline | -0.1 | -0.3 to 0.2 | 0.64 | -0.1 | -0.4 to 0.2 | 0.42 | -0.1 | -0.6 to 0.4 | 0.67 | -0.2 | -0.7 to 0.2 | 0.33 |
| KOOS_4_ at baseline | -0.4 | -0.8 to 0.1 | 0.10 | -0.0 | -0.5 to 0.5 | 0.91 | **-0.4** | **-1.3 to 0.5** | **0.41** | -0.5 | -1.3 to 0.3 | 0.24 |
| Rehabilitation visits | 0.0 | -0.1 to 0.1 | 0.93 | -0.0 | -0.2 to 0.1 | 0.81 | 0.2 | -0.1 to 0.4 | 0.17 | 0.1 | -0.1 to 0.4 | 0.22 |

**Supplementary Appendix Table 2:** Crude (unadjusted) results for participants treated with delayed ACL reconstruction (n=30)

Effect (regression coefficient): the estimate of the average change in a KOOS subscale (scores range from 0 (worst) to 100 (best)) that corresponds to a 1-unit change in the prognostic factor (1-unit = 1-point on a 0 to 100 scale for KOOS_4_ and SF-36 MCS)

Non-ACL surgery: total number of knee surgery events *not* performed in the same surgical session as ACL reconstruction or revision

KOOS and SF-36 scores range from 0 (worst) to 100 (best)

MCS: Mental Component Score

KOOS_4_: a mean score from four Knee injury and Osteoarthritis Outcome Score subscales (Pain, Symptoms, Sport/Rec and QOL)

ACLR: anterior cruciate ligament (ACL) reconstruction

**Statistically significant effects (p value <0.05) are highlighted in bold**

|  | **KOOS-Pain** | | | **KOOS-Symptoms** | | | **KOOS-Sport-Rec** | | | **KOOS-QOL** | | |
| --- | --- | --- | --- | --- | --- | --- | --- | --- | --- | --- | --- | --- |
|  | Crude effect | 95% CI | p | Crude effect | 95% CI | p | Crude effect | 95% CI | p | Crude effect | 95% CI | p |
| Baseline cartilage defect | -2.1 | -12.1 to 8.0 | 0.70 | -4.3 | -18.0 to 9.4 | 0.52 | -3.6 | -22.6 to 15.3 | 0.70 | -4.3 | -24.3 to 15.7 | 0.66 |
| Baseline meniscus damage | -6.9 | -17.1 to 3.3 | 0.18 | -10.1 | -23.9 to 3.7 | 0.15 | -5.6 | -25.3 to 14.1 | 0.57 | -16.9 | -36.8 to 3.0 | 0.09 |
| Baseline osteochondral lesion | -4.8 | -13.7 to 4.1 | 0.28 | -4.4 | -16.6 to 7.8 | 0.46 | -6.3 | -23.1 to 10.5 | 0.45 | 2.3 | -15.6 to 20.3 | 0.79 |
| Baseline extension deficit | -2.2 | -11.4 to 6.9 | 0.62 | -6.1 | -18.4 to 6.2 | 0.32 | -2.6 | -19.8 to 14.6 | 0.76 | -4.0 | -22.2 to 14.1 | 0.65 |
| Graft/contralateral ACL rupture | - | - | - | - | - | - | - | - | - | - | - | - |
| No non-ACL surgery | **8.7** | **0.3 to 17.2** | **0.04** | **12.9** | **1.7 to 24.2** | **0.03** | **16.3** | **0.5 to 32.1** | **0.04** | **21.7** | **5.9 to 37.6** | **0.01** |
| 1 non-ACL surgery | -7.3 | -16.7 to 2.1 | 0.12 | -7.5 | -20.5 to 5.5 | 0.25 | -11.7 | -29.4 to 6.1 | 0.19 | -5.6 | -24.9 to 13.6 | 0.55 |
| ≥2 non-ACL surgeries | -3.3 | -13.8 to 7.2 | 0.52 | -8.7 | -22.7 to 5.2 | 0.21 | -8.4 | -28.0 to 11.1 | 0.38 | **-22.8** | **-41.7 to -3.9** | **0.02** |
| SF-36 MCS at baseline | **0.3** | **0.1 to 0.6** | **0.01** | **0.4** | **0.1 to 0.8** | **0.01** | 0.3 | -0.1 to 0.8 | 0.15 | 0.4 | -0.1 to 0.9 | 0.07 |
| KOOS_4_ at baseline | 0.2 | -0.1 to 0.6 | 0.18 | 0.2 | -0.3 to 0.7 | 0.45 | 0.4 | -0.2 to 1.1 | 0.21 | 0.3 | -0.4 to 1.0 | 0.32 |
| Rehabilitation visits | -0.1 | -0.3 to 0.1 | 0.39 | -0.0 | -0.3 to 0.2 | 0.77 | -0.2 | -0.5 to 0.2 | 0.32 | -0.3 | -0.7 to 0.1 | 0.10 |

**Supplementary Appendix Table 3:** Crude (unadjusted) results for participants treated with exercise-therapy alone (n=29)

Effect (regression coefficient): the estimate of the average change in a KOOS subscale (scores range from 0 (worst) to 100 (best)) that corresponds to a 1-unit change in the prognostic factor (1-unit = 1-point on a 0 to 100 scale for KOOS_4_ and SF-36 MCS)

Non-ACL surgery: total number of knee surgery events *not* performed in the same surgical session as ACL reconstruction or revision

KOOS and SF-36 scores range from 0 (worst) to 100 (best)

MCS: Mental Component Score

KOOS_4_: a mean score from four Knee injury and Osteoarthritis Outcome Score subscales (Pain, Symptoms, Sport/Rec and QOL)

ACLR: anterior cruciate ligament (ACL) reconstruction

**Statistically significant effects (p value <0.05) are highlighted in bold**
